# Soft Computing in Medical Image Processing

**DOI:** 10.1155/2016/7358162

**Published:** 2016-07-12

**Authors:** Syoji Kobashi, László G. Nyúl, Jayaram K. Udupa

**Affiliations:** ^1^Graduate School of Engineering, University of Hyogo, 2167 Shosha, Himeji, Hyogo 6712280, Japan; ^2^Institute of Informatics, University of Szeged, P.O. Box 652, Szeged 6701, Hungary; ^3^Department of Radiology, University of Pennsylvania, 3710 Hamilton Walk, 6th Floor, Philadelphia, PA 19104, USA

Recent advances in medical imaging modalities such as magnetic resonance (MR) imaging and multidetector computed tomography (MDCT) enable us to acquire high-dimensional, sectional, thin-sliced, and a large number of images within a short acquisition time. The sheer volume of data poses great challenges for human interpretation of images by radiologists. Image processing becomes essential to analyze such complex data.

Soft computing has been introduced into medical image processing because it is an effective approach to handle uncertainties inherent in acquired image data. Some examples in the past 20 years are fuzzy connectedness approaches to image segmentation, fuzzy clustering methods particularly for human brain MR image segmentation, and statistical atlases and fuzzy models for object recognition and delineation. Soft computing approaches include fuzzy logic, neural networks, support vector machines, evolutional computation, probabilistic approaches, and chaos theory.

This special issue aims to showcase recent advances in soft computing approaches in medical image processing. In response to the call for papers, we received 26 submissions from all over the world. All manuscripts underwent a very rigorous peer review process. We finally selected 8 full papers for this special issue.

Image segmentation plays an important role in medical image processing. L. Ma et al. propose a new method which combines artificial fish swarm algorithm and fuzzy c-means and demonstrates the performance on brain MR images. K. B. Kim et al. propose a contour extraction method for segmenting deep cervical flexor (DCF) muscles in ultrasonic images. The method is based on fuzzy sigma binarization process. L. Zhao and K. Jia introduce multiscale convolutional neural network (CNN) to brain tumor segmentation. Y. Li et al. show a method for segmenting white blood cells using a dual-thresholding approach.

Image fusion is also an effective computational methodology that is hard for a human to deal with. It combines images from two or more different modalities and generates a new image. G. Yang et al. propose a new method that fills nonsubsampled contourlet transform (NCST) coefficients using generalized Gaussian density. It has been applied to CT/MRI, MRI/positron emission tomography (PET) and MRI/PET image fusion, and the results are superior to conventional NCST-based methods.

In addition, there are unique medical image processing schemes that have been proposed. Jing and Sheng introduce a method to retrieve a 3D model from freehand sketch. It may be very useful for medical education, informed consent for patients, and so on. B. J. Park et al. propose a gesture based interface, and it can be utilized in the operating room where surgeons cannot touch computer interface devices such as mouse and keyboard during operation. L. Huang et al. show a dense trajectory and classification method on echocardiogram video images.

Each paper contributes an improvement of medical image processing in each application area, and also the approaches are applicable to other areas. We hope that the papers will enrich the readers' knowledge of biomedical image analysis and will in turn lead them to the exploration of new advances and avenues.

*Syoji Kobashi* *Syoji Kobashi* *László G. Nyúl* *László G. Nyúl* *Jayaram K. Udupa* *Jayaram K. Udupa*

